# DAP5 drives translation of specific mRNA targets with upstream ORFs in human embryonic stem cells

**DOI:** 10.1261/rna.079194.122

**Published:** 2022-10

**Authors:** Maya David, Tsviya Olender, Orel Mizrahi, Shira Weingarten-Gabbay, Gilgi Friedlander, Sara Meril, Nadav Goldberg, Alon Savidor, Yishai Levin, Vered Salomon, Noam Stern-Ginossar, Shani Bialik, Adi Kimchi

**Affiliations:** 1 Department of Molecular Genetics, Weizmann Institute of Science, Rehovot 7610001, Israel; 2 The Broad Institute, Cambridge, Massachusetts 02141, USA; 3 The Mantoux Bioinformatics Institute, Weizmann Institute of Science, Rehovot 7610001, Israel; 4 The de Botton Institute for Protein Profiling of the Nancy and Stephen Grand Israel National Center for Personalized Medicine (G-INCPM), Weizmann Institute of Science, Rehovot 7610001, Israel

**Keywords:** DAP5, ribosome profiling, RNA-seq, uORF, pluripotent embryonic stem cells, noncanonical protein translation

## Abstract

Death associated protein 5 (DAP5/eIF4G2/NAT1) is a member of the eIF4G translation initiation factors that has been shown to mediate noncanonical and/or cap-independent translation. It is essential for embryonic development and for differentiation of embryonic stem cells (ESCs), specifically its ability to drive translation of specific target mRNAs. In order to expand the repertoire of DAP5 target mRNAs, we compared ribosome profiles in control and DAP5 knockdown (KD) human ESCs (hESCs) to identify mRNAs with decreased ribosomal occupancy upon DAP5 silencing. A cohort of 68 genes showed decreased translation efficiency in DAP5 KD cells. Mass spectrometry confirmed decreased protein abundance of a significant portion of these targets. Among these was KMT2D, a histone methylase previously shown to be essential for ESC differentiation and embryonic development. We found that nearly half of the cohort of DAP5 target mRNAs displaying reduced translation efficiency of their main coding sequences upon DAP5 KD contained upstream open reading frames (uORFs) that are actively translated independently of DAP5. This is consistent with previously suggested mechanisms by which DAP5 mediates leaky scanning through uORFs and/or reinitiation at the main coding sequence. Crosslinking protein–RNA immunoprecipitation experiments indicated that a significant subset of DAP5 mRNA targets bound DAP5, indicating that direct binding between DAP5 protein and its target mRNAs is a frequent but not absolute requirement for DAP5-dependent translation of the main coding sequence. Thus, we have extended DAP5's function in translation of specific mRNAs in hESCs by a mechanism allowing translation of the main coding sequence following upstream translation of short ORFs.

## INTRODUCTION

Eukaryotic translation initiation factor 4 gamma 2 (eIF4G2, hereafter referred to as death associated protein 5 [DAP5]) is a member of the eIF4G translation initiation factors (Imataka et al. 1997; Levy-Strumpf et al. 1997; Shaughnessy et al. 1997; Yamanaka et al. 1997), and an RNA binding protein that emerged in several unbiased screens of the RNA interactome (Baltz et al. 2012; Castello et al. 2012; Kwon et al. 2013). eIF4G1 acts as a scaffold for assembly of the preinitiation complex at the 5′ cap structure, which recruits the 40S ribosome and other translation factors necessary for cap-dependent translation. DAP5 lacks the amino terminus of eIF4G1, including the domain that interacts with the cap-recognition factor eIF4E, and thus cannot mediate canonical cap-dependent translation. It can, however, modulate translation in a cap-independent manner by several mechanisms that have been proposed in the recent literature. DAP5 can recruit the ribosome to internal sequences within the 5′UTR known as the internal ribosome entry site (IRES), or cap-independent translation enhancers (CITE), thereby driving cap-independent translation of select mRNAs both in cell-free assays and intact cells (Weingarten-Gabbay et al. 2014; Liberman et al. 2015; Yoffe et al. 2016; Haizel et al. 2020). This mechanism has been shown to drive specific noncanonical translation, specifically in scenarios when general cap-dependent translation is attenuated, such as during apoptosis and stress conditions (e.g., c-Myc, Apaf-1, c-IAP2, and p53 [Henis-Korenblit et al. 2002; Nevins et al. 2003; Warnakulasuriyarachchi et al. 2004; Lewis et al. 2008; Weingarten-Gabbay et al. 2014]), mitosis (e.g., Bcl-2 and CDK1 [Marash et al. 2008]), and in pluripotent hESCs (e.g., HMGN3 [Yoffe et al. 2016]). A similar cap-independent translation mechanism that requires DAP5 recognizes *N*^6^-methyladenosine (m6A) modification of motifs adjacent to start codons within circular mRNAs. The m6A reader YTHDF3A, which can interact with DAP5, is required for translation of these methylated circular mRNAs, and is presumed to recruit DAP5 and eIF3A to the methylated motif (Yang et al. 2017). Another mechanism, demonstrated in quiescent mammalian cells and immature *Xenopus* oocytes, is mediated by sequences within the 3′UTR of specific mRNAs, via interactions between DAP5 and an FXR1a-miRNP complex that binds the mRNA (Bukhari et al. 2016). DAP5 was also shown to mediate a noncanonical, eIF4E-independent translation of capped mRNAs by binding eIF3d (de la Parra et al. 2018), which can directly interact with the 5′ cap structure to enable assembly of the initiation complex in the absence of eIF4E (Lee et al. 2016). DAP5/eIF3D-dependent translation was shown to be a widespread mechanism in breast cancer cells that may account for translation of nearly 20% of mRNAs (de la Parra et al. 2018). This mechanism also mediates expression of regulatory T cell differentiation-specific mRNAs following mTORC1 inhibition and TGFβ-induced transcriptional reprogramming of naïve T cells, during which eIF4E/cap-dependent translation is inhibited (Volta et al. 2021).

More recently, DAP5 was shown to mediate translation of the main coding sequences (CDS) in mRNAs containing structured 5′ leaders with upstream ORFs (uORFs) (Weber et al. 2021; Smirnova et al. 2022). Both works used reporter assays to prove that the 5′ leader harboring the uORFs was critical for translation of the main CDS in a DAP5-dependent manner. The uORFs, which were translated in a canonical cap-dependent manner, were the springboard to facilitate DAP5-mediated initiation from downstream translation start sites, including the CDS, and more distal uORFs. Thus, in contrast to the noncanonical mechanisms described above, this DAP5-mediated function indirectly requires the canonical eIF4G1 translation initiation complex that interacts with the 5′ cap. According to Weber et al. (2021), DAP5 reinitiates translation of the CDS by post-termination 40S subunits that failed to recycle following translation of the uORFs, whereas Smirnova et al. favored a mechanism by which DAP5 promotes leaky scanning through the uORF (Smirnova et al. 2022).

DAP5 function is critical for early development. Deletion of the DAP5 encoding gene *Eif4g2* by knockout (KO) in mice leads to early embryonic lethality at the gastrulation stage (Yamanaka et al. 2000). Similarly, knockdown (KD) of the zebrafish ortholog is embryonic lethal and results in impaired mesoderm formation (Nousch et al. 2007). Embryonic lethality was also observed in loss-of-function mutants of the *Drosophila* ortholog, which was shown to be necessary for germband extension. Mutants with a milder hypomorphic allele died in the early pupa stage during metamorphosis, exhibiting defective salivary gland regression, head egression, and adult sensory organ formation (Yoshikane et al. 2007). In cell culture, KO of *Eif4g2* in mouse embryonic stem cells (mESCs) and KD of DAP5 in human ESCs (hESCs) negatively affected their ability to differentiate in response to various triggers such as retinoic acid (RA), or when grown as embryoid bodies (EBs) or teratomas (Yamanaka et al. 2000; Yoffe et al. 2016).

Importantly, we previously reported that hESCs depleted of DAP5 by shRNA exhibited a reduced ability to drive cap-independent translation (Yoffe et al. 2016), supporting the hypothesis that DAP5's translation functions are necessary for embryonic differentiation. Analysis of the set of mRNAs that exhibited reduced association with heavy polysomes, that is, reduced translation efficiency (TE) in DAP5 KD hESCs, indicated that DAP5 is necessary for translation of ribosomal proteins and proteins involved in mitochondrial respiration, which is critical for the transition from pluripotency to differentiation (Yoffe et al. 2016). The same study also showed that DAP5 mediates cap-independent translation of *HMGN3* mRNA, which encodes a chromatin remodeling factor that is necessary for RA-induced differentiation (Yoffe et al. 2016). Ribosome profiling in *Eif4g2* KO mESCs indicated that DAP5 is necessary for the translation of MAP3K3 and Sos1; consistent with this, *Eif4g2* KO mESCs had reduced ERK signaling, thus resembling ground state mESCs (Sugiyama et al. 2017). Altogether, the cohort of DAP5 target mRNAs necessary for the transition of ESCs from pluripotency to differentiated states may be larger than initially assumed and should be explored further.

Here, we wished to expand the list of DAP5-dependent mRNA translation targets and uncover additional mechanisms of noncanonical translation in hESCs. To this end, we performed ribosome profiling together with mass spectrometry analysis in DAP5 KD hESCs, identifying an interesting set of mRNAs with reduced TE and corresponding reduced protein abundance. We further characterized this set of mRNAs by determining which bound directly to DAP5 by a genome-wide RNA-coimmunoprecipitation (CLIP) assay. Notably, a significant portion of the DAP5 target mRNAs contained uORFs within their 5′ leader sequences; DAP5 KD reduced translation from the main coding sequence but not from cap-proximal uORFs, supporting the hypothesis that DAP5 is necessary for reinitiation and/or leaky scanning of target mRNAs in hESCs.

## RESULTS AND DISCUSSION

### Ribosome profiling identifies translation targets of DAP5 in hESCs

**FIGURE 1.**
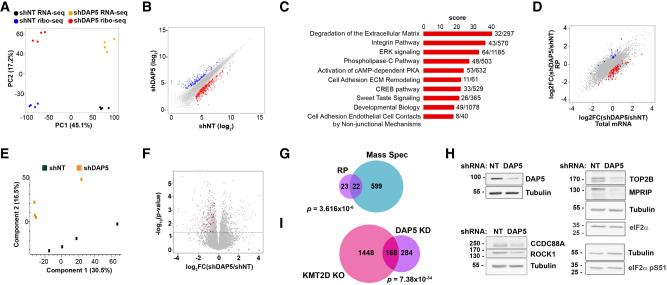
Ribosome profiling identifies mRNAs with reduced translation efficiency upon DAP5 KD in hESCs. (*A*) Principal component analysis (PCA) of transcript profiles of DAP5-KD (shDAP5) and NT (shNT) hESCs Ribo-seq and total RNA-seq, from four independent experiments. (*B*) Scatter plot of total mRNA transcript expression in DAP5-KD and NT hESCs, on a log_2_ scale. Transcripts with decreased abundance in DAP5-KD hESCs (FC < 0.5, FDR < 0.05) are highlighted in red, and transcripts with increased abundance (FC > 2, FDR < 0.05) are highlighted in blue. (*C*) Pathway analysis by GeneAnalytics of differentially expressed genes identified by RNA-seq of total mRNA upon KD of DAP5 in hESCs, compared to control NT KD. Scores are based on *P*-values, after correction for multiple comparisons by the false discovery rate (FDR) method; top 10 high quality score pathways are listed. Numbers at *right* correspond to the number of matched genes out of the total number of genes in the pathway. (*D*) Comparative analysis of the translation efficiency (TE) in DAP5-KD and NT hESCs. Genes are plotted as a scatter plot according to changes in ribosome occupancy (RP) on the *y*-axis and mRNA abundance (total mRNA) on the *x*-axis. The values are shown on a log_2_ scale. Each dot represents an individual gene (*n* = 14,511). Genes without significant changes in TE are indicated in gray. Genes with increased or decreased TE (FC > 1.5-fold or <0.67-fold, *P*-value < 0.001) are highlighted in blue (16 genes) and red (68 genes), respectively. (*E*) PCA of MS analysis of DAP5 and control NT KD hESCs, from four independent experiments. (*F*) Volcano plot showing protein abundance as determined by MS analysis of DAP5 KD and NT KD hESCs, expressed as log_2_(fold-change). Dashed line indicates *P* < 0.05, all dots *above* the line showed a significant change upon DAP5 depletion. Proteins with significant decreased abundance that were identified as DAP5 activated translation targets in the ribosome profiling screen are colored in red. (*G*) Venn diagram showing the overlap between the set of significantly decreased proteins identified by MS, and the set of translationally activated mRNAs that were detected by RP. (*H*) Western blotting was performed on lysates from shNT and shDAP5 hESCs to detect protein levels of DAP5 and potential DAP5 translation targets, as indicated. Shown are representative blots from one of three (two for MPRIP) experiments. Tubulin was used as a loading control on each of the four blots shown. I. Venn diagram showing overlap between set of mRNAs down-regulated upon KO of *Kmt2d* in mESCs and KD of DAP5 in hESCs. Note that sequence coverage was much larger in the former experiment; only genes detected in both screens were included. *P*-values for overlap in G and I were calculated by hypergeometric test.

As previously reported, DAP5 KD pluripotent hESCs did not exhibit any differences in proliferation or survival rates compared to control cells, and global translation rates were unchanged (Yoffe et al. 2016). Yet TE of specific proteins was affected by DAP5 depletion in pluripotent hESCs, as assessed by reduced association of these specific mRNAs with polysomes in DAP5 depleted hESCs. Ribosome profiling (RP) is a second method that recognizes actively translated mRNAs by virtue of their occupancy by ribosomes and thus protection from RNase treatment. The protected sequences are subsequently identified by deep sequencing (Ribo-seq). Since polysome profiling and ribosome profiling often detect different subsets of translation targets, we performed RP-Ribo-seq in hESCs expressing DAP5 shRNA or control nontargeting (NT) shRNA as an alternative strategy to identify DAP5-specific translation targets. RP has the advantage in that it quantitatively pinpoints which regions of the mRNA are bound by ribosomes, including actively translated uORFs with potential regulatory functions. In parallel, RNA deep sequencing (RNA-seq) was also performed on total mRNA, and the ribosome loading score (RLS) of each mRNA was determined by normalization of ribosome footprints to its total mRNA abundance as a measure of TE (Finkel et al. 2021a). The effect of DAP5 depletion on the RLS of each mRNA was calculated by comparing control NT and DAP5 KD hESCs (Supplemental Table S1). Principle component analysis (PCA) indicated that both Ribo-seq and RNA-seq experiments were reproducible, as replicates clustered together (Fig. 1A). Metagene analysis of read densities, in which gene profiles are aligned and then averaged, revealed the expected profiles of footprints in the Ribo-seq experiment (Supplemental Fig. S1A). Analysis of the transcriptome of DAP5 KD cells yielded 493 down-regulated mRNA transcripts (fold-change < 0.5, FDR < 0.05) and 225 up-regulated mRNA transcripts (fold-change > 2.0, FDR < 0.05) (Fig. 1B; Supplemental Table S1). GeneAnalytics pathway analysis of the down-regulated group indicated enriched terms associated with extracellular matrix (ECM) and cellular adhesion, such as “degradation of extracellular matrix,” “integrin pathway,” and “cell adhesion ECM remodeling,” and signaling pathways involving ERK, PKA PLC, and CREB (Fig. 1C). The former is particularly interesting considering the importance of ECM and integrin interactions for ESC differentiation and cell fate (Wang et al. 2015), and that ESC-derived ECM affects their differentiation in response to different cues (Sart et al. 2014). “Developmental biology” was also a highly enriched pathway, and several gene ontology (GO) terms related to development, especially of the nervous system, were enriched within the data set (Supplemental Fig. S1B). There were no highly enriched pathways within the set of up-regulated genes. Upon normalization of the RP and total mRNA data, a group of 68 genes showed a significant reduction in TE (>1.5-fold, *P* < 0.001) upon depletion of DAP5; these mRNAs require DAP5 for their translation either directly or indirectly (Table 1; Fig. 1D; Supplemental Table S1). This set will hereafter be referred to as DAP5 translationally activated mRNAs. A smaller cohort of genes (*n* = 16) showed increased RLS in the DAP5-KD cells compared to the NT control (Fig. 1D; Supplemental Table S1).

**TABLE 1.**
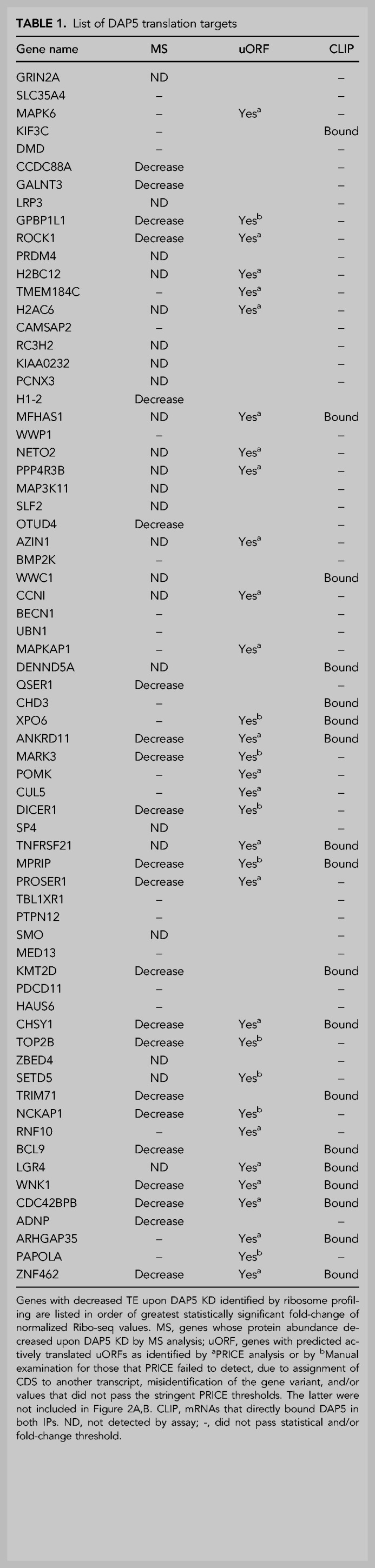
List of DAP5 translation targets

The group of DAP5 translationally activated mRNAs was too small for meaningful GO analysis. However, manual annotation indicated that this group contained several cytoskeletal signaling enzymes and cytoskeletal interacting proteins, including three serine/threonine kinases: ROCK1 (Rho associated protein kinase-1), CDC42BPB (CDC42 binding protein kinase beta), and MARK3 (microtubule affinity regulating kinase 3), the phosphatase MPRIP (myosin phosphatase Rho interacting protein), the actin binding protein CCDC88A (coiled-coil domain containing 88A), the microtubule-organizing protein, CAMSAP2 (calmodulin regulated spectrin associated protein family member 2), NCKAP1, a protein that regulates actin filament reorganization via its interaction with the Arp2/3 complex, and ARHGAP35 (Rho GTPase activating protein 35), which modulates Rho GTPase-dependent F-actin polymerization. Other serine/threonine kinases with different functions, such as MAPK6, BMP2K (BMP2 inducible kinase), and WNK1, are also part of this DAP5 translationally activated cohort. Three genes, WNK1, MARK3, and MPRIP, were also identified as DAP5 translation targets in the previously reported RP analysis of mESCs (Yamanaka et al. 2000), which described a relatively small set of 13 mRNAs (excluding DAP5 itself) with reduced TE in the DAP5 KO (23% overlap, *P* < 2.722 × 10^−5^).

In order to validate whether the identified DAP5-activated mRNA translation targets show reduced protein steady-state levels upon DAP5 KD, and to confirm that the RLS calculated in the Ribo-seq analysis indeed reflects TE, mass spectrometry (MS) was performed in control and DAP5 KD hESCs. PCA indicated that although there was some variation among the four replicates from each group, the samples clustered according to the KD group, indicating good reproducibility in the response to DAP5 silencing (Fig. 1E). Overall, of the 68 encoded proteins from the cohort of mRNAs with reduced TE, only 45 were detected by MS. Notably, the expression of 22 of these 45 proteins was significantly reduced in the DAP5 KD cells compared to the NT KD control (*P* < 0.05), confirming the RP results (Table 1; Fig. 1F, red dots, 1G; Supplemental Table S2). The remainder did not show a significant change. Obviously, as additional levels of regulation dictate the steady-state abundance of a protein, such as regulation of protein turnover, a full overlap with changes in TE is not expected. To confirm the MS data, western blotting was performed on control and DAP5 KD hESC lysates for selected proteins for which efficient antibodies were available. As predicted, KD of DAP5 led to reductions in the steady-state protein levels of CCDC88A, ROCK1, TOP2B, and MPRIP (Fig. 1H). Thus, the western blots confirm the reduced abundance observed for these proteins in the MS data, consistent with reduced translation of their mRNAs upon depletion of DAP5, as indicated by the Ribo-seq analysis.

Changes in the steady-state levels of the remaining proteins in the MS data set that showed no change in TE (gray dots in Fig. 1F; 675 and 602 proteins with decreased and increased abundance, respectively) are the indirect consequence of the DAP5 KD. Notably, these yielded a relatively weak correlation with changes in the mRNA steady-state levels (RNA-seq), which implies that they are mainly regulated at the protein level (Supplemental Fig. S1C,D). It may also reflect the inconsistent and sometimes incomplete correlation between RNA-seq and MS data due to the very different methodologies used, each with different biases, sensitivity and coverage (Wang et al. 2019; Buccitelli and Selbach 2020). Notably, no significant changes in abundance were observed for the canonical translation initiation factors that were detected by MS (Supplemental Table S2, e.g., eIF2 [α, β, and γ subunits], eIF3 subunits, eIF4GI, eIF4E, eIF5). Furthermore, western blotting confirmed that neither eIF2α (eIF2S1) levels, nor its phosphorylation on Ser51, were affected by DAP5 KD (Fig. 1H). Thus, changes in TE observed in the RP analysis are likely due to DAP5's direct effects on its targets, rather than indirect effects resulting from changes in expression of other translation factors.

Among the DAP5 translationally activated mRNAs that showed decreased protein abundance in the MS analysis is the histone methylase *KMT2D* (*MLL4*). KMT2D, a member of the Set1/COMPASS (COMplex of Proteins ASsociated with Set1) family of methyltransferases, mono-methylates Histone H3 on Lys4 within nucleosomes associated with enhancers. KMT2D is necessary for embryonic development and functions as a tumor suppressor. In mESCs, *Kmt2d* KO is not necessary for self-renewal and expression of pluripotent genes but is necessary for exit from naïve ground state to primed pluripotency (Cao et al. 2018). It is also necessary for differentiation of mESCs into EBs and is required for gene expression associated with the differentiated phenotype (Wang et al. 2016). As the major H3 methylase in mESCs, KMT2D enhancer priming is specifically required for binding of the p300 acetylase and subsequent activation of genes required during differentiation (Cao et al. 2018). Notably, the phenotype of *Kmt2d* KO in mESCs greatly resembles that of *Eif4g2* KO in mESCs (Sugiyama et al. 2017) and of DAP5 KD in hESCs (Yoffe et al. 2016). Thus, it is likely that as a DAP5 translation target, KMT2D is important for mediating part of the differentiation defect that results from DAP5 deficiency. In fact, a comparison of the set of differentially expressed genes in *Kmt2d* KO mESCs with the RNA-seq of total mRNA in the DAP5 KD hESCs indicated a significant overlap between the two sets of down-regulated genes, consisting of 168 genes out of the 452 genes showing reduced mRNA expression upon DAP5 KD that were also detected in the former screen (Fig. 1I). This overlap is all the more remarkable considering that KMT2D was but one of many DAP5 translationally activated targets identified in our RP screen, and that its reduced TE upon DAP5 KD resulted in a partial decrease in abundance at the protein level, far from the complete deletion obtained by KO in the comparison set. Thus, although not excluding additional critical targets, the necessity for DAP5-dependent control of KMT2D protein expression is likely a contributing factor to the differentiation defects observed upon DAP5 perturbation in ECSs.

### Identification of uORFs within DAP5 mRNA targets

**FIGURE 2.**
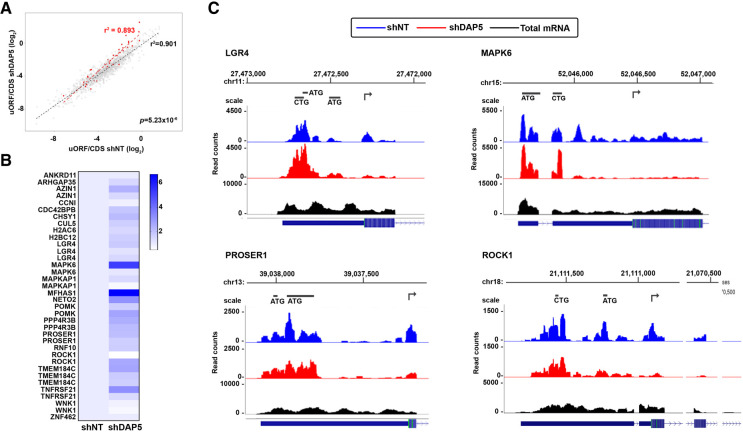
uORFs within DAP5 translationally activated mRNAs are translated independently of DAP5. (*A*) A correlation between the uORF read count of the shNT samples and the shDAP5 samples as calculated by PRICE. Each data point represents an uORF, where the uORF read count was normalized to the read count of the main CDS. DAP5 translationally activated transcripts are colored in red filled circles. The dashed red and black lines are the linear regression fit to DAP5 activated uORFs and the remaining uORFs, respectively. Statistical significance between the two groups was determined by the ANCOVA test. (*B*) Heat map of the mean uORF/CDS ratio in control and DAP5 KD hESCs for DAP5 translationally activated targets identified by PRICE. Mean ratios were obtained from the uORF and CDS reads of the four Ribo-seq replicates, and were normalized to the mean shNT ratio, expressed as 1. Color scale indicates increasing mean uORF/CDS ratio, indicative of increasing DAP5-dependent translation of CDS as compared to uORF. uORFs with similar or lower mean ratios in DAP5 KD hESCs compared to NT hESCs correspond to uORFs that are likewise translated in a DAP5 dependent manner. Targets with more than one uORF identified are presented accordingly with individual uORF/CDS ratios. (*C*) Ribosome profiling footprints in NT (blue) and DAP5 (red) KD hESCs, and total mRNA reads (black, one representative NT KD sample) distribution along the 5′ leader and the 5′ proximal coding sequence of the indicated genes. Shown are snapshots from the UCSC Genome Browser with the predicted uORFs demarked by black lines of corresponding lengths with initiation codon indicated. Ribo-seq replicates are presented in track collection mode using merging method “add.” The structure of the mRNA is shown at the *bottom*. Chromosomal position is shown at *top*. For clarity, all genes are shown in the 5′ to 3′ orientation regardless of the chromosomal orientations of their respective loci; genes that are encoded on the minus strand are shown on the reverse strand (e.g., LGR4, PROSER1, ROCK1). Genes for which the 5′UTRs span more than a single exon are presented using “multi-exon” view. Arrows indicate initiation of CDS. Additional genes with manually detected uORFs that were missed by the PRICE platform are shown in Supplemental Figure S2.

In light of recent reports showing that DAP5 mediates reinitiation at the main CDS following translation of uORFs in HEK293 cells (Weber et al. 2021), and/or leaky ribosome scanning through the uORFs in NIH3T3 fibroblasts (Smirnova et al. 2022), the DAP5 mRNA targets identified here were analyzed for the presence of uORFs to determine if this mechanism is relevant to hESCs. To this end, the RP results were analyzed using PRICE (probabilistic inference of codon activities by an EM algorithm). This computational method relies on a combination of translation features to predict actively translated ORFs and codons from RP measurements (Erhard et al. 2018). After applying FDR < 0.05 correction, 2691 uORFs and 6850 CDS (including 3280 truncated ORFs) were detected. These were further filtered to include only transcripts in which the annotated CDS was successfully captured by PRICE and at least one uORF was detected in the 5′ UTR (>10 reads in the NT or DAP5 KD samples), yielding 1195 uORFs (Supplemental Table S3). The ratio between the number of reads mapped to uORFs and the CDS on the same transcript was then computed and compared between the DAP5 and NT KD samples. Of the 68 DAP5 translationally activated genes identified by RP, the PRICE platform recognized 36 uORFs corresponding to 23 different CDSs (Table 1; Supplemental Table S3). Thus, according to the stringent PRICE criteria, 33.8% of DAP5 translationally activated target mRNAs contained at least one uORF. This was a significant enrichment compared to the remaining 14,443 mRNAs, of which 924 contained uORFs that were recognized by PRICE and passed our filters (Supplemental Table S3, 6.4%, *P* < 1.0 × 10^−5^ by χ^2^ statistical test). Most importantly, calculations of the ratio between the number of reads mapped to uORFs and the CDS indicated that overall, DAP5 translationally activated mRNAs showed a higher uORF/CDS ratio in the DAP5-KD samples compared to the NT control KD samples (Fig. 2A,B). The significant increase in uORF/CDS ratios indicates a relative accumulation of ribosomes on the uORFs in comparison to CDSs specifically in the DAP5 KD cells, implying reduced translation of the CDS but not uORF upon DAP5 depletion. This is consistent with the proposed mechanisms by which DAP5 is required for reinitiation and/or leaky read-through following uORF translation in these mRNA targets. Figure 2C shows ribosome footprints of several representative mRNAs with this trend. Peaks corresponding to sequences to which ribosomes bound are indicated. In both NT and DAP5 KD hESCs, such peaks are observed in the 5′ UTR leader, corresponding to uORFs. However, ribosome binding peaks are reduced on the CDSs in the DAP5 KD cells. Most targets had multiple uORFs, represented by separate peaks. The ones more proximal to the 5′ cap showed DAP5-independent ribosome occupancy, presumably utilizing cap-dependent eIF4G1 translation initiation complexes, while uORFs that were in close proximity to the main ATG were often DAP5-dependent, similar to the CDS (e.g., MAPK6 and ROCK1, Fig. 2B,C).

Since the PRICE pipeline by its stringent nature does not identify all uORFs (note that only 6% of the detected mRNAs were predicted by PRICE to contain uORFs, when previous approximations have estimated the number of genes with uORFs as ∼50% [Chen and Tarn 2019]), manual examination of the potential DAP5 mRNA targets that were missed was performed by a visual examination of the RP footprints, noting peaks that corresponded to regions identified as uORFs by PRICE. This analysis yielded an additional nine mRNAs with uORFs that were excluded from the original PRICE results due to misidentifications of the transcript or to stringency of the filters (i.e., did not pass the FDR threshold) (Table 1; Supplemental Table S3). The RP footprints for five of these genes are shown in Supplemental Figure S2. Manual observation of the RP footprint of WNK1, which contained two uORFs identified by PRICE that showed DAP5-dependent ribosome binding (Fig. 2B), revealed additional cap-proximal uORFs that were translated independently of DAP5 (Supplemental Fig. S2). Thus, a total of 32 of 68 DAP5 mRNA targets (47%) are predicted to be regulated by DAP5 via a reinitiation or leaky scanning mechanism on the CDS following uORF translation, the latter of which is DAP5 independent. Of note, these targets were missed in our previously reported polysome analysis of DAP5-dependent translation (Yoffe et al. 2016), perhaps due to the presence of ribosomes on uORFs; since the polysome-associated fractions were pooled and compared to free ribosome fractions, this method did not detect shifts to lighter polysome-bound fractions that would reflect reduced translation from the CDS while maintaining ribosomal binding to uORFs. Thus, the RP method currently used has an advantage over polysomal profiling as it provides independent measurements of individual ORFs encoded from a single transcript, and has the resolution to probe differential effects of translation initiation factors on these individual ORFs.

### Identification of mRNAs that directly bind DAP5

**FIGURE 3.**
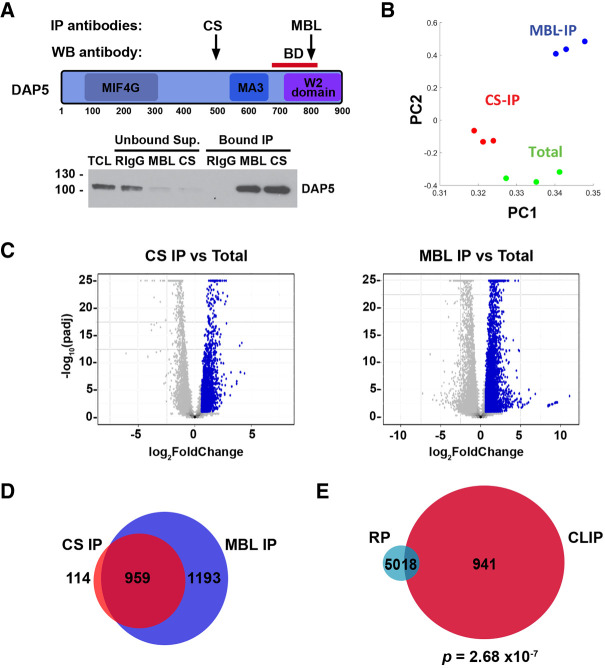
CLIP-seq identifies mRNAs that bind DAP5. (*A, top*) Schematic representation of DAP5 domain organization. Locations of epitopes of the MBL and CS anti-DAP5 antibodies that were used for IP are indicated with black arrows. Red bar indicates the location of the epitope of the mouse monoclonal anti-DAP5 antibody that was used for western blotting. (*Bottom*) Western blot of total cell lysate (TCL) and DAP5 IP with the two rabbit anti-DAP5 antibodies. Rabbit IgG (RIgG) was used as a control. (*B*) Principal component analysis (PCA) of transcript profiles of the two IPs, each performed in triplicate, and total mRNA from the same experiments, showing the variability among the samples. The two components of the PCA explain 98% of the variance among the samples. (*C*) Volcano plots of DAP5 CLIP-seq results from RNA IP using CS antibody (*left*) and MBL antibody (*right*). Blue dots indicate mRNAs that were enriched in the DAP5 bound fraction compared to the total RNA (Fold-change > 2, FDR < 0.05). (*D*) Venn diagram comparing the overlap between the sets of DAP5 bound mRNAs identified in the two IPs. (*E*) Venn diagram comparing the overlap between the set of DAP5 bound mRNAs identified by CLIP and the set of DAP5 translationally activated mRNA targets identified by RP. The overlap was statistically significant, as determined by cumulative distribution function (CDF) of the hypergeometric distribution.

To investigate whether DAP5's translation function on CDS downstream from uORFs requires direct binding to the corresponding mRNAs, a genome-wide cross-linking immuno-precipitation (CLIP) assay was conducted in the parental hESCs. Specifically, protein–RNA complexes were cross-linked by UV in intact cells, followed by immunoprecipitation (IP) using two antibodies directed against different epitopes in the DAP5 protein (Fig. 3A). Total mRNA and the bound mRNA were then subjected to deep sequencing. The IP samples obtained from each of the different antibodies were compared to the total mRNA for fold-change calculation. The CLIP assay was performed in triplicate, with strong reproducibility among the experiments, as indicated by PCA analysis (Fig. 3B). Genes with a fold-change above two compared to their levels in the total mRNA, with an adjusted *P*-value <0.05, were considered to be enriched in the DAP5 bound samples (Fig. 3C, blue dots). A comparison of the bound samples obtained with the two different antibodies illustrated a high degree of overlap, corresponding to 959 mRNAs common to both antibodies (out of 1073 and 2152 enriched mRNAs identified by CS and MBL antibodies, respectively) (Fig. 3D; Supplemental Table S4). Of the 68 DAP5 translation targets identified by RP, 18 mRNAs (26.5%) directly bind to DAP5 protein according to the CLIP data, a statistically significant enrichment (*P* < 2.68 × 10^−7^, Table 1; Fig. 3E; Supplemental Table S4). These included 11 of the 32 mRNAs that harbor uORFS (34%), and an additional seven that did not, including *KMT2D* (Table 1; Supplemental Table S4). Thus, it appears that direct binding of DAP5 protein to its mRNA targets often characterizes the way in which DAP5 mediates translation initiation, although the binding sites on these mRNA targets has not yet been mapped.

In conclusion, we have identified a group of direct DAP5 translation targets in hESCs, characterized by a 5′UTR containing uORFs that are translated independently of DAP5, while DAP5 promotes translation of the main CDS downstream from these uORFs. These comprise nearly half of all DAP5-translationally activated mRNAs identified in our screen. The presence of uORFs often suppresses translation of the downstream CDS, and is subjected to tight regulation, for example, under stress conditions when global translation is constrained (Chen and Tarn 2019). Notably, the cellular milieu of pluripotent ESCs shares characteristics of cell stress, including high levels of eIF2α Ser51 phosphorylation and reduced global translation (Sampath et al. 2008; Friend et al. 2015). DAP5 specifically facilitates translation of its target mRNAs in hESCs, overcoming the potential suppressive effect of the uORFs. Although phospho-eIF2α can promote translation of mRNAs containing uORFs (i.e., yeast *GCN4* and mammalian *ATF4* and *GADD34*) (Young and Wek 2016), DAP5 depletion did not affect basal eIF2α phosphorylation, implying that DAP5's effects occur through other mechanisms. It is likely that this involves physical association of DAP5 with its target mRNAs. A significant portion of the translationally activated mRNAs directly bind DAP5, although this is not an absolute requirement for DAP5-dependent translation of the main CDS. We postulate that indirect binding through additional RNA binding factors may facilitate DAP5-dependent initiation of the main CDS in these mRNAs. Significantly, KO of DAP5 in HEK293T cells also led to decreased TE of (at least) two DAP5 targets identified here, WNK1 and ROCK1, both of which contain translated uORFs (Weber et al. 2021). Using reporters containing the 5′ leader mRNA of WNK1, Weber et al. demonstrated that DAP5 drives reinitiation of the downstream start codon at the main CDS following translation of the uORFs. Thus, it is likely that a similar mechanism functions to drive translation of WNK1 in hESCs, and by extension, additional DAP5-dependent targets bearing uORFs. Notably, DAP5 has also been proposed to facilitate leaky scanning through the uORFs (Smirnova et al. 2022); our data does not discriminate between these mechanisms.

As not all of the identified DAP5 targets contain uORFs, additional DAP5-dependent mechanisms exist to drive translation of other mRNA targets in hESCs, as suggested by our previous work (Yoffe et al. 2016). Furthermore, the identification of mRNA targets whose TE increased upon DAP5 KD suggests an additional level of negative regulation by DAP5 on protein translation. One such mechanism was recently described for the dipeptide repeat protein encoded by G_4_C_2_ repeats within the first intron of *C9ORF72*, which is negatively regulated by DAP5-dependent translation of an uORF within the first exon (van ‘t Spijker et al. 2022). Thus, by expanding the repertoire of DAP5 mRNA targets in hESCs, we have promoted further mechanistic insight into the mode of action of DAP5-dependent translation.

## MATERIALS AND METHODS

### Cell culture

hESC H9 (Wa-09) cells were maintained on a feeder layer of iMEFs (irradiated mouse embryonic fibroblasts) in hESC medium (DMEM F-12, 20% knockout serum [Thermo Fisher], 1% NEAA, 0.5% glutamine, 0.1 mM β-mercaptoethanol, 8 ng/mL bFGF). The cells were grown for one passage before transfer to Matrigel-coated plates containing mTeSR (STEMCELL Technologies) for experiments. The medium was changed daily for hESCs grown in either condition. For CLIP, H9 cells were detached using StemPro Accutase (Thermo Fisher) and replated on Matrigel-coated plates.

Stable KD of DAP5 was generated by infecting H9 hESCs with lentiviruses harboring pLKO.1-puro plasmid expressing shRNA to DAP5 (Sigma TRCN0000147914) or nontargeting control shRNA (NT), followed by selection using puromycin, as previously described. DAP5 KD cells were fully viable and exhibited no growth defects (Yoffe et al. 2016).

### Preparation of samples for ribosome profiling and RNA-seq

For RNA-seq, control (NT) and KD hESCs were washed with PBS and then collected with Tri-Reagent (Sigma-Aldrich), total RNA was extracted, and poly(A) selection was performed using Dynabeads mRNA DIRECT Purification Kit (Invitrogen). mRNA samples were subjected to DNase I treatment and 3′ dephosphorylation using FastAP Thermosensitive Alkaline Phosphatase (Thermo Scientific) and T4 PNK (NEB) followed by 3′ adaptor ligation using T4 ligase (NEB). The ligated products were used for reverse transcription with SSIII (Invitrogen) for first-strand cDNA synthesis. The cDNA products were 3′ ligated with a second adaptor using T4 ligase and amplified with eight cycles of PCR for final library products of 200–300 bp. For Ribo-seq libraries, cells were subsequently treated with 100 µg mL^−1^ CHX for 1 min. Cells were then placed on ice, washed twice with PBS containing 100 µg mL^−1^ CHX, scraped from 10 cm plates, pelleted and lysed with lysis buffer (1% Triton X-100 in 20 mM Tris pH 7.5, 150 mM NaCl, 5 mM MgCl_2_, 1 mM dithiothreitol supplemented with 10 U mL^−1^ Turbo DNase and 100 µg mL^−1^ CHX). After lysis, samples stood on ice for 2 h and subsequent Ribo-seq library generation was performed as previously described (Finkel et al. 2021b). In brief, cell lysate was treated with RNaseI for 45 min at room temperature followed by SUPERase-In quenching. The sample was loaded on sucrose solution (34% sucrose, 20 mM Tris pH 7.5, 150 mM NaCl, 5 mM MgCl_2_, 1 mM dithiothreitol, and 100 µg mL^−1^ CHX) and spun for 1 h at 100,000 rpm in a TLA-110 rotor (Beckman) at 4°C. The pellet was resuspended in TRI reagent and the RNA was collected using chloroform phase separation. For size selection, 15 µg total RNA was separated on a 15% TBE-urea gel for 65 min, and 28–34 footprints were excised using 28 and 34 flanking RNA oligos, followed by RNA extraction and Ribo-seq protocol (Finkel et al. 2021b). Total RNA-seq and Ribo-seq libraries were sequenced on an Illumina NovaSeq 6000 system.

### Ribosome profiling and RNA-seq analysis

Paired-end reads were first trimmed from their linker sequence (CTGTAGGCACCATCAAT) and poly(A) using cutadapt and filtered from rRNA reads by bowtie (v1.2.3) alignment to rRNA library. Reads that passed this filtration were aligned to human genome version hg38 using STAR version 2.7.3a. Only uniquely aligned reads were used for further analyses. For total RNA, normalization of the counts and differential expression analysis was performed using DESeq2 (version 1.30.1) (Love et al. 2014) using default parameters. Raw *P*-values were adjusted for multiple testing using the procedure of Benjamini and Hochberg. Genes with a fold-change ≥2/≤0.5, with a *P*_adj_ ≤ 0.05 and average normalized read count of total RNA samples >10 were considered as differentially expressed genes. For analysis of total RNA from *Kmt2d* KO mESCs, raw data were downloaded from GEO accession GSE99022 (Cao et al. 2018), and analyzed as above. For Ribo-seq analysis, reads were counted in the coding region excluding 15 and 6 nt from the initiating methionine and ends of each CDS, respectively (Ingolia et al. 2009; McGlincy and Ingolia 2017). The read count normalization and *P*-values were calculated with DEseq2 using a model that included the genotype (shDAP5 and shNT) and sample type (Ribo-seq, RNA-seq) as contrasts and an interaction term between the two. Ribosome loading score (RLS) was measured as log_2_(RP/Total), and the fold change upon DAP5 KD was calculated from the ratio of RLS (shDAP5) to RLS (shNT). Genes with fold-changes of at least 1.5, with a *P*-value <0.001, and base mean >10 were considered as genes with significant change in their RLS. RLS was assumed to reflect TE, as later confirmed by observed decreases in protein steady-state levels.

### Prediction of translation initiation sites

Translation initiation sites and uORF identification were predicted using PRICE (Erhard et al. 2018), a computational method that models experimental noise to enable researchers to accurately resolve noncanonical translation initiation and short ORFs. PRICE was run using hg38 and GENCODE V38. The results were filtered to include transcripts with at least 10 average read count in either the shNT samples or the shDAP5 samples. This was applied for the uORFs (ORF types: uORF, uoORF) and main CDSs (ORF types: CDS, Trunc). Transcripts for which both uORF and main ORF predictions passed our filters, and with FDR < 0.05, were considered as transcript with uORFs.

### CLIP and deep RNA-sequencing

hESC H9 (Wa-09) cells grown on Matrigel were UV cross-linked at 0.15 mJ/cm^2^ (254 nm). Cells were harvested and snap frozen. The cell pellets were resuspended in lysis buffer (50 mM Tris–HCl, pH 7.4, 100 mM NaCl, 1% NP-40, 0.1% SDS, 0.5% sodium deoxycholate, with protease inhibitors) and treated with Turbo DNase (Ambion). Equal amounts of protein were used (2 mg). Rabbit anti-DAP5 antibodies (Cell Signaling, 5169 or MBL, RN003P) or control rabbit IgG (Sigma) were precoated onto Protein-A Dynabeads (Invitrogen). DAP5–RNA complexes were immunoprecipitated at 4°C for 2 h, followed by stringent washes, with up to 1 M NaCl. The bound RNA was detached from the beads by proteinase K (New England BioLabs, P8107S), and extracted using phenol:chloroform standard procedures. In parallel, total RNA was extracted from 100 ng of the cell lysate. Three biological replicas of immunoprecipitated and total RNA were processed using the SMARTer Ultra Low Input RNA Kit protocol (Clontech) according to the manufacturer's instructions. Libraries were evaluated by Qubit and Bioanalyzer. Sequencing libraries were constructed with barcodes to allow multiplexing of all samples on one lane. The sequencing was run on an Illumina HiSeq 2000 instrument. The obtained reads, Single-Read 60 bp long, were trimmed using cutadapt and mapped to the human genome (hg38) using STAR v2.4.2a (default parameters). Median sequencing depth was ∼19 million reads per sample and fairly homogenous across samples. Approximately 98% reads were mapped to the genome and ∼90% of the uniquely mapped reads were counted (∼16,500 mRNA transcripts). Counting proceeded over genes annotated in RefSeq, using htseq-count (intersection-strict mode). Differential bound analysis was performed using DESeq2 (version 1.10.1) with the betaPrior, cooksCutoff, and independentFiltering parameters set to False. Raw *p*-values were adjusted for multiple testing using the procedure of Benjamini and Hochberg. Pipeline was constructed using Snakemake. The cutoff for bound genes was a fold-change of at least two compared to the total RNA, FDR < 0.05 and average normalized counts of at least 50.

### Mass spectrometry

For sample preparation, four samples from DAP5-KD and four from NT control hESCs were subjected to in-solution tryptic digestion using the S-Trap method (by Protifi). The resulting peptides were fractionated offline using high pH reversed phase chromatography, followed by online nanoflow liquid chromatography (nanoAcquity) coupled to high resolution, high mass accuracy mass spectrometry (Thermo Orbitrap Exploris 480). Samples from each fraction were analyzed on the instrument separately, and within each fraction samples were analyzed in random order in discovery mode. The raw data were processed with MaxQuant v1.6.6.0. The data was searched with the Andromeda search engine against the human and mouse proteome databases as downloaded from Uniprot.org, appended with common laboratory protein contaminants and the following modifications: fixed modification-cysteine carbamidomethylation; variable modifications-methionine oxidation, asparagine and glutamine deamidation, and protein amino-terminal acetylation. The quantitative comparisons were calculated using Perseus v1.6.0.7. Decoy hits were filtered out, and only proteins that were detected in at least two replicates of at least one experimental group were kept. Contamination signals from the mouse proteome, resulting from initial coculture on MEF feeder cells, was estimated to be approximately two orders of magnitude weaker than the human proteome signal, based on a quantitative index called iBAQ, and mouse genes were filtered out from the data.

### Western blot analysis

shNT and shDAP5 hESCs were lysed with RIPA buffer, and 50 µg lysate was resolved on 8.5% SDS-PAGE gels for western blotting, according to standard protocols. The following primary antibodies were used: DAP5 (BD Biosciences, cat# 610742); CCDC88A (Cell Signaling, cat# 14200); MPRIP (Proteintech, cat# 20040-1-AP), ROCK1 (Cell Signaling, cat# 4035T) TOP2B (Proteintech, cat# 20549-1-AP), eIF2α (Santa Cruz, cat# sc11386), phoshoSer51 eIf2α (Abcam, cat# ab32157), Tubulin (Sigma-Aldrich, cat# T9026). Secondary antibodies were HRP-conjugated anti-mouse or anti-rabbit IgG (Jackson ImmunoResearch cat# 111-035-06 or cat# 115-035-003).

### Statistical analysis

Statistical tests were applied as indicated in figure legends and/or text.

## DATA DEPOSITION

All RNA sequencing data for total RNA (RNA-seq) and Ribo-seq experiments in NT and DAP5 KD cells and CLIP-seq experiments in H9 cells were deposited in NCBI GEO Gene Expression Omnibus, accession GSE193115. The mass spectrometry proteomics data have been deposited in the ProteomeXchange Consortium via the PRIDE (Perez-Riverol et al. 2019) partner repository with the data set identifier PXD030717.

## SUPPLEMENTAL MATERIAL

Supplemental material is available for this article.

## Supplementary Material

Supplemental Material

## Figures and Tables

**Maya David:**
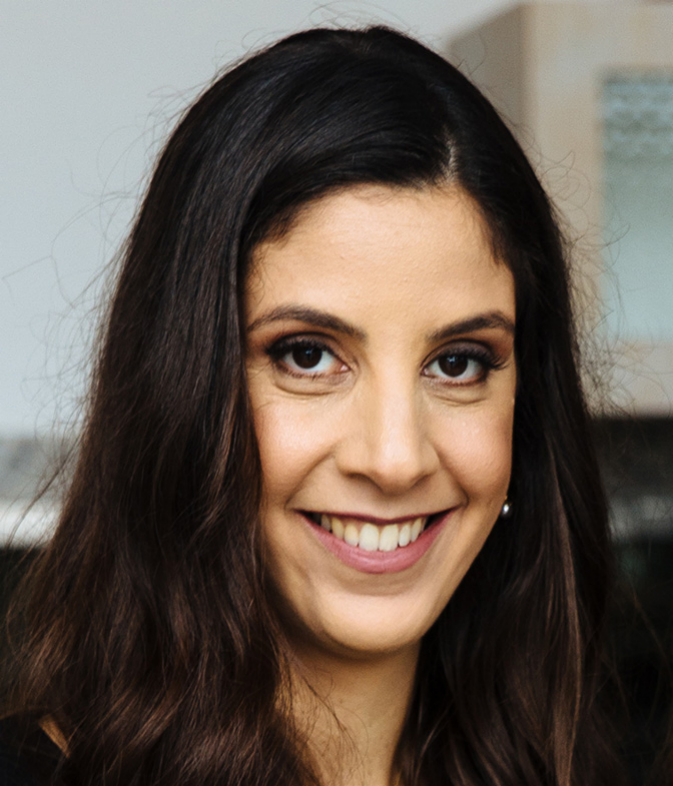


## MEET THE FIRST AUTHOR

***Meet the First Author(s)* is a new editorial feature within *RNA*, in which the first author(s) of research-based papers in each issue have the opportunity to introduce themselves and their work to readers of *RNA* and the RNA research community. Maya David is the first author of this paper, “DAP5 drives translation of specific mRNA targets with upstream ORFs in human embryonic stem cells.” Maya is a former PhD student and a post-doc fellow in Professor Adi Kimchi's laboratory at the Weizmann Institute of Science, who studied the role of DAP5 in translation regulation during human embryonic stem cell differentiation.**

**What are the major results described in your paper and how do they impact this branch of the field?**

In this paper, we showed that DAP5, which is a noncanonical translation initiation factor, promotes translation of the main CDS in mRNAs containing translated uORFs in human embryonic stem cells. By applying global ribosomal profiling, protein mass spectrometry, and CLIP assays, we have extended the cohort of DAP5 translated mRNA targets and learned more about its mode of action in hESCs.

**What led you to study RNA or this aspect of RNA science?**

I was always fascinated by RNA and translation regulation. When I started my PhD, there was an increased interest in translation control during embryonic development. DAP5, a well-studied protein in my laboratory, has an essential role in embryonic differentiation, driving exit from pluripotency. However, not much was known on the molecular mechanism and its mRNA targets in human embryonic stem cells.

**If you were able to give one piece of advice to your younger self, what would that be?**

Always keep an open mind and be led by the results. Analyze the data in an unbiased way and investigate it carefully. You can discover things that you never thought of.

**Are there specific individuals or groups who have influenced your philosophy or approach to science?**

Being a scientist, you face a lot of challenges and some disappointments. My mentor Professor Adi Kimchi, with her optimistic approach, taught me to look at the big picture and that there is always something new that we can learn.

**What are your subsequent near- or long-term career plans?**

I have decided to take my interest in RNA into real life implications. Recently, I started working for SKIP Therapeutics, which develops RNA-based treatments for rare genetic diseases using splice modulating antisense oligonucleotides (ASOs).
