# Supplementary material for: DAP5 drives translation of specific mRNA targets with upstream ORFs in human embryonic stem cells
Source: RNA. 2022 Oct;28(10):1325–36. doi: 10.1261/rna.079194.122 (PMC9479741; doi:10.1261/rna.079194.122)
Supplement: Supplemental Material [file supp_28_10_1325__DC1.html]

DAP5 drives translation of specific mRNA targets with upstream ORFs in human embryonic stem cells — Supplemental Material 

# DAP5 drives translation of specific mRNA targets with upstream ORFs in human embryonic stem cells

## Supplemental Material

- Supplemental\_Fig\_S1.pdf
- Supplemental\_Fig\_S2.pdf
- Supplemental\_Legends.docx
- Supplemental\_Table\_Legends.docx
- Supplemental\_Table\_S1.xlsx
- Supplemental\_Table\_S2.xlsx
- Supplemental\_Table\_S3.xlsx
- Supplemental\_Table\_S4.xlsx
