# Supplementary material for: DAP5 drives translation of specific mRNA targets with upstream ORFs in human embryonic stem cells
Source: RNA. 2022 Oct;28(10):1325–36. doi: 10.1261/rna.079194.122 (PMC9479741; doi:10.1261/rna.079194.122)
Supplement: Supplemental Material [file supp_079194.122_Supplemental_Fig_S1.pdf]

Supplemental Figure S1

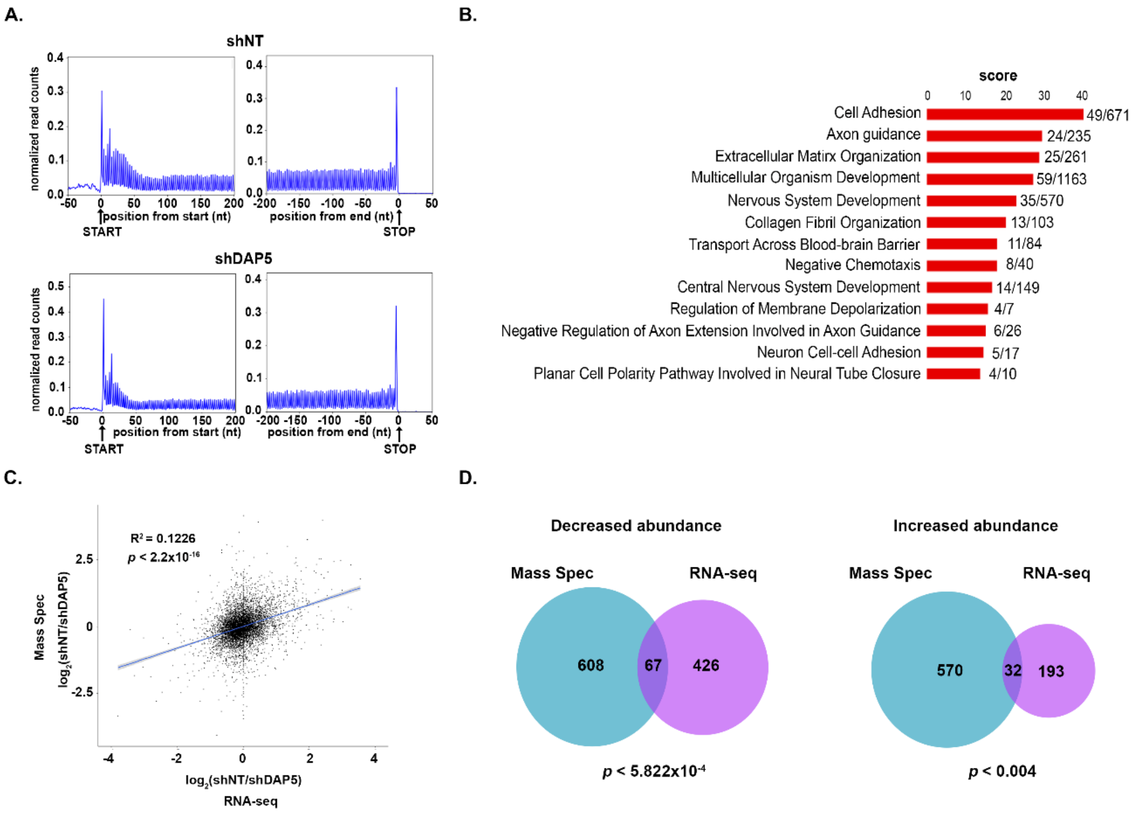

Supplemental Figure S1. Analysis of Ribo-seq and RNA-seq datasets. A. Metagene analysis of read densities relative to the maximal signal of each gene around the start and stop codons of cellular CDSs at 5 hpi. Shown are representative plots of all 4 repeats for shNT and shDAP5 samples. **B.** Enriched GO terms as determined by GeneAnalytics of differentially expressed genes identified by RNA-seq of total mRNA upon KD of DAP5 in hESCs, compared to control NT KD. Scores are based on *p*-values, after correction for multiple comparisons by the false discovery rate (FDR) method; only high quality score GO terms are listed. Numbers at right correspond to the number of matched genes out of the total number of genes in the pathway. **C.** Correlation between differentially expressed genes identified in RNA-seq and proteins with changed abundance as identified by MS. Dots represent the derived fold-change of each gene in either dataset, line represents the calculated linear regression. **D.** Venn diagrams showing overlap between RNA-seq and MS datasets, for transcripts/proteins with decreased abundance and increase abundance, excluding those that were shown to be DAP5 translation targets. Statistical significance was determined by hypergeometric test.
