# Supplementary material for: DAP5 drives translation of specific mRNA targets with upstream ORFs in human embryonic stem cells
Source: RNA. 2022 Oct;28(10):1325–36. doi: 10.1261/rna.079194.122 (PMC9479741; doi:10.1261/rna.079194.122)
Supplement: Supplemental Material [file supp_079194.122_Supplemental_Fig_S2.pdf]

Supplemental Figure S2

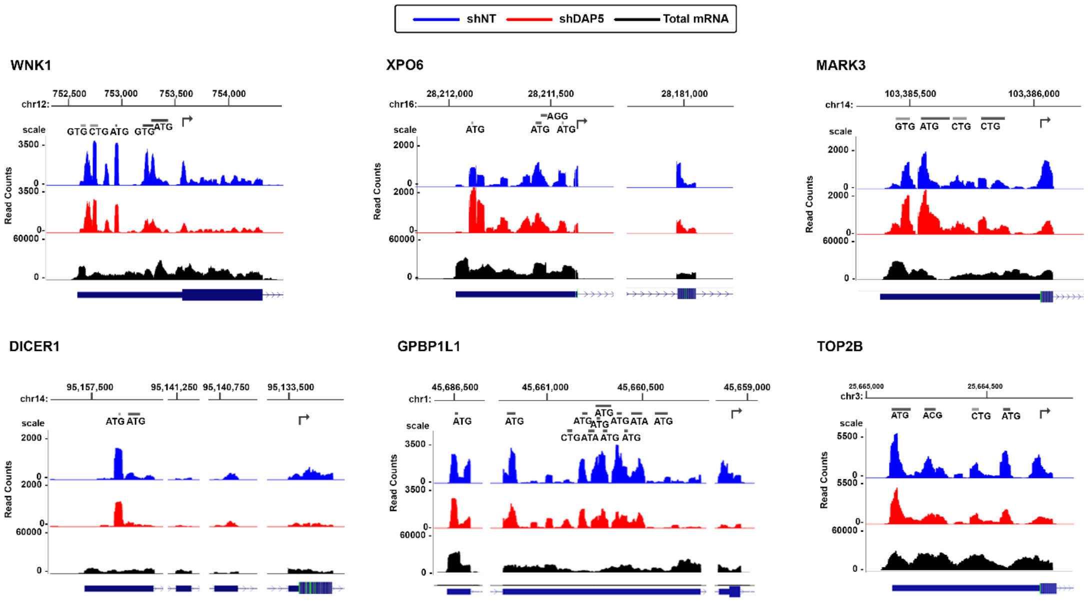


**Supplemental Figure S2. DAP5 translationally activated genes are enriched with uORFs.** Ribosome profiling footprints (blue, red) and total mRNA reads (black, one representative NT KD sample) distribution along the 5′ leader and the 5′ proximal coding sequence of the indicated genes, in NT and DAP5 KD hESCs. Shown are snapshots from the UCSC genome browser, with Ribo-Seq replicates presented in track collection mode using merging method "add". The structure of the mRNA is shown at the bottom. Chromosomal positions are shown at top. For clarity, all genes are shown in the 5' to 3' orientation regardless of the chromosomal orientations of their respective loci; genes that are encoded on the minus strand are shown on the reverse strand (i.e. XPO6, DICER1, GPBP1L1 and TOP2B). Genes for which the 5'UTRs span more than a single exon (DICER1, GPBP1L1) are presented using "multi exon" view with chromosomal segments split accordingly. Arrows indicate initiation of CDS. Note that for XPO6, the exon containing the 5'UTR contains the very beginning of the CDS, which continues in a distant exon. Dark grey bars indicate position and length of manually detected, statistically significant uORFs ($p<0.05$) uORFs that were nevertheless omitted from the PRICE analysis due to misidentification/misclassification of the corresponding CDS. Note that these did not necessarily pass the more stringent FDR threshold used for the PRICE-identified uORFs. Positions and lengths of non-significant uORFs ($p>0.05$, as defined by PRICE criteria) are also indicated by light grey bars. Start codon of each uORF is indicated next to the corresponding bar. Note that the PRICE-recognized uORFs in the WNK1 gene proximal to the CDS (black bars) are DAP5 dependent, while the manually identified uORFs proximal to the 5' end of the mRNA are DAP5 independent. Further details on manually detected uORFs can be found in Supplementary Table S3, third tab.
