# Supplementary material for: DAP5 drives translation of specific mRNA targets with upstream ORFs in human embryonic stem cells
Source: RNA. 2022 Oct;28(10):1325–36. doi: 10.1261/rna.079194.122 (PMC9479741; doi:10.1261/rna.079194.122)
Supplement: Supplemental Material [file supp_079194.122_Supplemental_Legends.docx]

**Supplemental Material**

Large datasets are presented as Excel tables (.xlxs) in Supplemental Materials with accompanying legends (Supplemental_Table_Legends.doc).

**Supplemental Table S1**, Results of Ribosome Profiling and DAP5 regulated mRNAs **Supplemental Table S2**, List of proteins with decreased levels upon DAP5 KD as identified by Mass Spectrometry

**Supplemental Table S3**, List of all uORFs identified in the Ribo-seq experiment using the PRICE tool

**Supplemental Table S4**, Results of CLIP analysis and DAP5 bound mRNAs

**Supplemental Figure S1**, Additional analysis of RNA-seq, MS and Ribo-seq experiments

**Supplemental Figure S2,** Ribosome footprints of DAP5 translationally activated target genes with manually detected uORFs
