# Supplementary material for: DAP5 drives translation of specific mRNA targets with upstream ORFs in human embryonic stem cells
Source: RNA. 2022 Oct;28(10):1325–36. doi: 10.1261/rna.079194.122 (PMC9479741; doi:10.1261/rna.079194.122)
Supplement: Supplemental Material [file supp_079194.122_Supplemental_Table_Legends.docx]

**Legends for Supplemental Tables**

**Supplemental Table S1. Results of Ribosome Profiling and DAP5 regulated mRNAs.**

First tab shows differentially expressed genes in DAP5 KD hESCs, defined as those with fold change <0.5 or >2.0, FDR <0.05. Second tab shows genes translationally regulated by DAP5, defined as those with a TE fold-change ratio >1.5, *p*<0.001 in shDAP5 vs control shNT KD hESCs. Genes highlighted in pink were downregulated by DAP5 KD, those in blue were up-regulated. Third tab shows all normalized total mRNA reads and the DEseq2 statistical analysis of the shDAP5 vs shNT samples. Fourth tab shows all normalized mRNA levels in ribosome profiling (RP) and total mRNA sequencing in each of 4 repeats for shDAP5 and shNT hESCs, and the DEseq2 analysis of the results.

**Supplemental Table S2. List of proteins with decreased levels upon DAP5 KD as identified by Mass Spectrometry.**

First tab shows the proteins whose levels significantly decreased (p<0.05) in shDAP5 vs shNT KD hESCs, as detected by MS analysis. Second tab shows all the results of the MS, with intensities shown per protein in each of 4 repeats for shDAP5 and shNT KD hESCs. RNA-seq data was also included as a comparison, for those genes that were detected in both methods.

**Supplemental Table S3. List of all uORFs identified in the Ribo-seq experiment using the PRICE tool.** First tab shows significant results after application of statistical filters. Genes identified as DAP5 activated in the Ribo-seq experiment are indicated by 1 in last column, and are also shown separately in the second tab. *P*-values were calculated by PRICE and were corrected for multiple testing using FDRtools package. Note that some genes are represented by more than 1 uORF. Types of PRICE predictions include: uoORF, upstream overlapping ORF starts in 5'-UTR and ends within CDS; uORF, upstream ORF starts and ends in 5'-UTR. CDSs are indicated as either CDS (ORF is exactly as in the annotation) or Trunc (ORF is contained in a CDS, ending at its stop codon). Ratios of reads from uORF and CDS sequences are presented for shNT and shDAP5 samples. Third tab shows PRICE data for genes in which uORFs were identified manually, as the tool assigned the CDS to a separate transcript or otherwise mis-identified the CDS, as indicated. ncRNA, non-coding RNA, identified as such instead of as uORF due to analysis of wrong cDNA variant.

**Supplemental Table S4. Results of CLIP analysis and DAP5 bound mRNAs.**

First tab shows list of mRNAs identified in CLIPs with both MLB and CS anti-DAP5 antibodies. Bound mRNAs were defined as those with base mean>50, fold-change ratios of levels in IP compared to total mRNA levels >2, and adjusted *p*<0.05. Final column indicates which of the bound proteins are also DAP5 translation targets, according to RP Ribo-seq data. Second tab shows all normalized data from each of three repeats from total mRNA, and IPs with MLB and CS anti-DAP5 antibodies.
